# A Review on Graphene-Based Gas/Vapor Sensors with Unique Properties and Potential Applications

**DOI:** 10.1007/s40820-015-0073-1

**Published:** 2015-11-26

**Authors:** Tao Wang, Da Huang, Zhi Yang, Shusheng Xu, Guili He, Xiaolin Li, Nantao Hu, Guilin Yin, Dannong He, Liying Zhang

**Affiliations:** 1 Key Laboratory for Thin Film and Microfabrication of Ministry of Education, Department of Micro/Nano Electronics, School of Electronic Information and Electrical Engineering, Shanghai Jiao Tong University, Shanghai, 200240 People’s Republic of China; 2 National Engineering Research Center for Nanotechnology, Shanghai, 200241 People’s Republic of China

**Keywords:** Graphene, Gas/Vapor sensor, Chemiresistor, Detection mechanism

## Abstract

Graphene-based gas/vapor sensors have attracted much attention in recent years due to their variety of structures, unique sensing performances, room-temperature working conditions, and tremendous application prospects, etc. Herein, we summarize recent advantages in graphene preparation, sensor construction, and sensing properties of various graphene-based gas/vapor sensors, such as NH_3_, NO_2_, H_2_, CO, SO_2_, H_2_S, as well as vapor of volatile organic compounds. The detection mechanisms pertaining to various gases are also discussed. In conclusion part, some existing problems which may hinder the sensor applications are presented. Several possible methods to solve these problems are proposed, for example, conceived solutions, hybrid nanostructures, multiple sensor arrays, and new recognition algorithm.

## Introduction

**Fig. 1:**
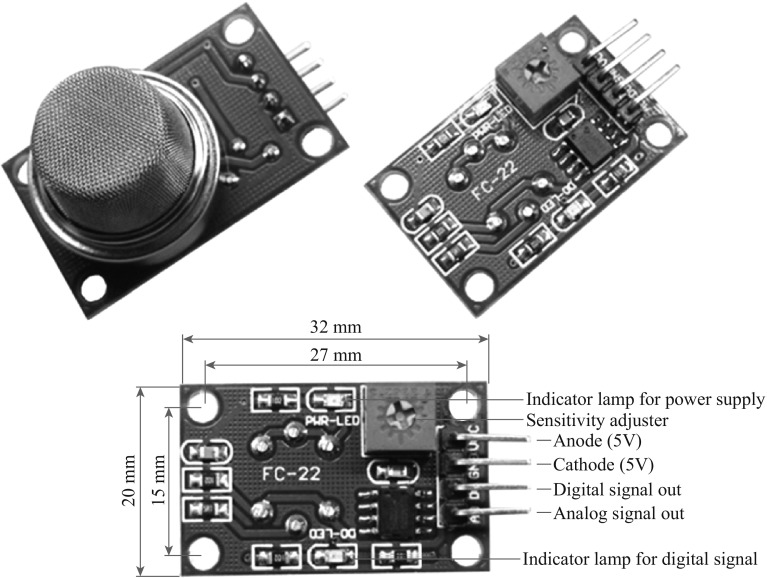
SnO_2_-based gas sensor for CO detection, product model: MQ-9

The past several decades have witnessed a tremendous development of chemical sensors in many fields [1–4]. Gases detecting and harmful vapors with early warning feature are playing increasingly important roles in many fields, including environmental protection, industrial manufacture, medical diagnosis, and national defense. Meanwhile, sensing materials are of intense significance in promoting the combination properties of gas/vapor sensors, such as sensitivity, selectivity, and stability. Thus, various materials [5–13], covering from inorganic semiconductors, metal oxides, and solid electrolytes, to conducting polymers, have been exploited to assemble sensing devices with small sizes, low power consumption, high sensitivity, and long reliability. Among them, nanomaterials, such as carbon nanotubes (CNTs), metal-oxide nanoparticles, and graphenes, are widely used in gas sensing for their excellent responsive characteristics, mature preparation technology, and low cost of mass production, since the traditional silicon-based semiconducting metal-oxide technologies will have reached their limits [14]. Figure 1 shows a module of MQ-9, a SnO_2_-based gas sensor for CO detection, which can be easily obtained in the market.

**Fig. 2:**
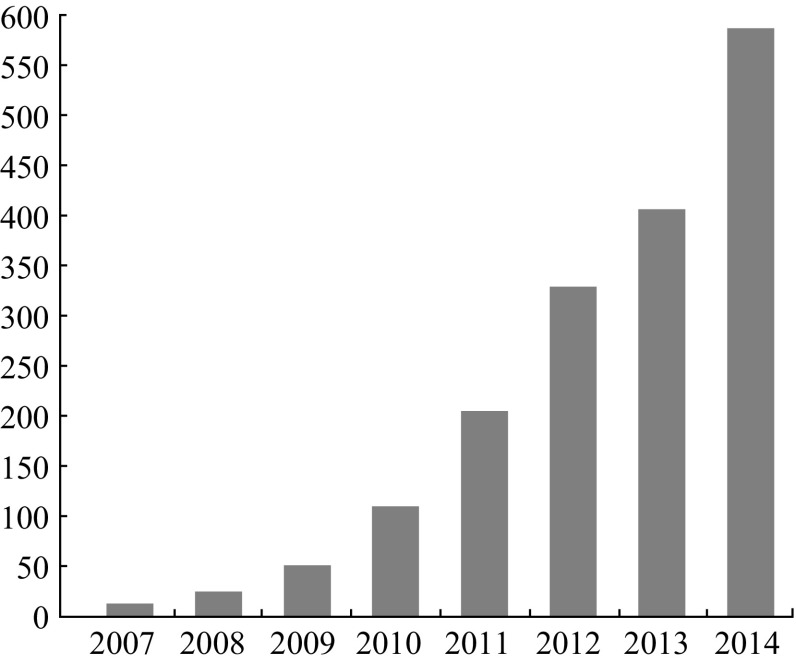
Histogram detailing the number of graphene-based gas/vapor sensors publications per year for the period from 2007 to 2014 (data obtained from ISI Web of Knowledge, January 28, 2015)

**Fig. 3:**
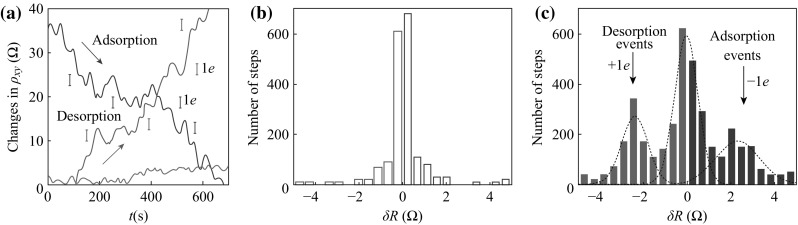
Single-molecule detection. **a** Examples of changes in Hall resistivity observed near the neutrality point (|*n*| < 10^11^ cm^−2^) during adsorption of strongly diluted NO_2_ (*blue curve*) and its desorption in vacuum at 50 °C (*red curve*). The *green curve* is a reference—the same device thoroughly annealed and then exposed to pure He. The *curves* are for a three-layered device in *B* = 10 T. The grid lines correspond to changes in *ρ*_*xy*_ caused by adding one electron charge, *e* (*δR* ≈ 2.5 Ω), as calibrated in independent measurements by varying *V*_g_. For the *blue curve*, the device was exposed to 1 ppm of NO_2_ leaking at a rate of ≈10^−3^ Ω mbar L s^−1^. Statistical distribution of step heights, R, in this device without its exposure to NO_2_ (in helium) (**b**) and during a slow desorption of NO_2_ (**c**). For this analysis, all changes in *ρ*_*xy*_ larger than 0.5 Ω and quicker than 10 s (lock-in time constant was 1 s making the response time of ≈6 s) were recorded as individual steps. The *dotted curves* in textbfc are automated Gaussian fits. Adapted from reference [24]. (Color figure online)

As one of the most fascinating materials, graphene has aroused scientists’ great enthusiasms in its synthesis, modification, and applications in many fields since 2004 [15], due to its remarkable overall properties, for instance, single-atom-thick two-dimensional conjugated structures, room-temperature stability, ballistic transport, and large available specific surface areas [16–39]. Graphene can be served as an ideal platform to carry other components for specific roles, because of its special structure. High conductivity and ballistic transport ensure that graphene exhibits very little signal disturbance when it works as a chemical sensor [40], which do not require auxiliary electric heating devices due to its excellent chemical stability at ambient temperature [16, 27]. All of these features for graphene are beneficial for its sensing properties, making it an ideal candidate for gas/vapor detecting. Therefore, great efforts have been put into the research of graphene-based gas/vapor sensors, leading to a giant leap in the development of graphene-based gas-sensing devices [24, 41–57]. We can clearly see that the number of published papers on graphene-based gas sensors has sharply increased over the period from 2007, as shown in Fig. 2. The first experiment focusing on the detection of gas molecules based on graphene was carried out in 2007. Schedin et al. reported that micrometer-size sensors made from graphene were capable of detecting single gas molecules attached to or detached from graphene’s surface, as depicted in Fig. 3 [24]. Their discovery indicated that graphene had a great potential for detecting and sensing.

**Fig. 4:**
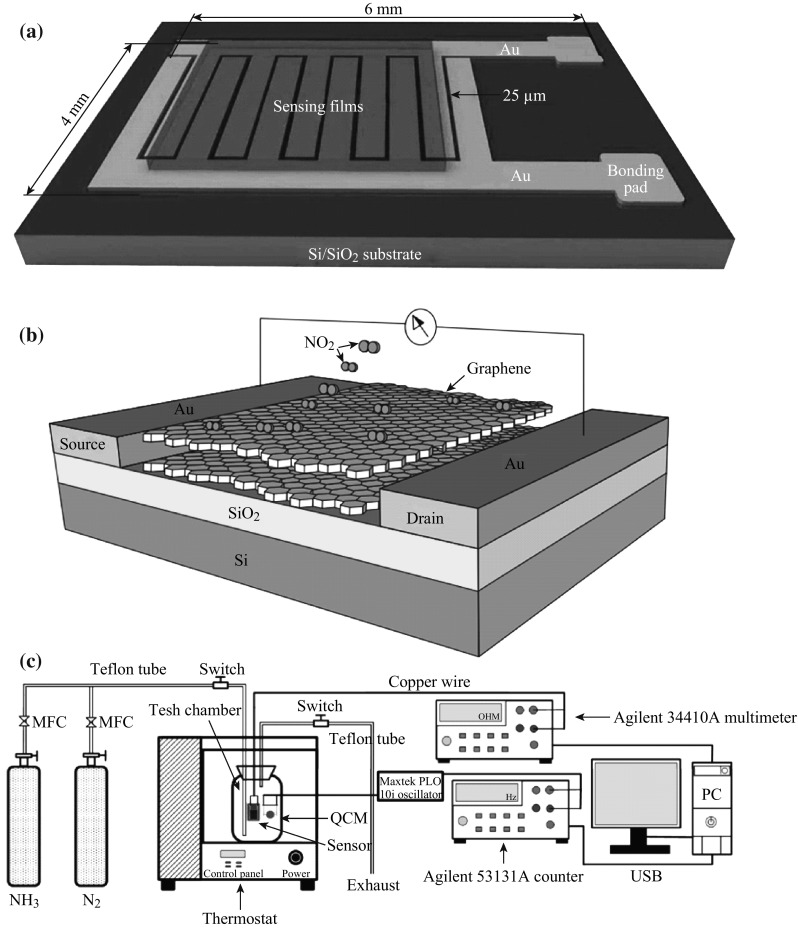
Typical schematic diagram of **a** chemiresistor, **b** FET, and **c** testing system of gas sensors with chemiresistor structure. Adapted from reference [61, 62]

In principle, a sensor is a device, purpose of which is to sense (i.e., to detect) some characteristics of its environs. It detects events or changes in quantities and provides a corresponding output, generally as an electrical or optical signal. According to different forms of reaction with external atmospheres, gas/vapor sensors can be classified into chemiresistor, silicon-based field-effect transistor (FET), capacitance sensor (CS), surface work function (SWF) change transistor, surface acoustic wave (SAW) change transistor, optical fiber sensor (OFS), and so on [58]. Among them, chemiresistor is the most widely used in the construction of gas/vapor sensors and also the most popular product for practical applications, because of its long-history research, simple structure, convenience to implement, room-temperature operation, and relatively low cost [59, 60]. Actually, we usually apply voltage on both electrodes of the device, and detect the current fluctuating over time when gas composition changes. Figure 4 distinctly shows the typical structure of chemiresistors and silicon-based FET devices. An ordinary testing system for the research of gas sensors with chemiresistor structure is also displayed.

**Fig. 5:**
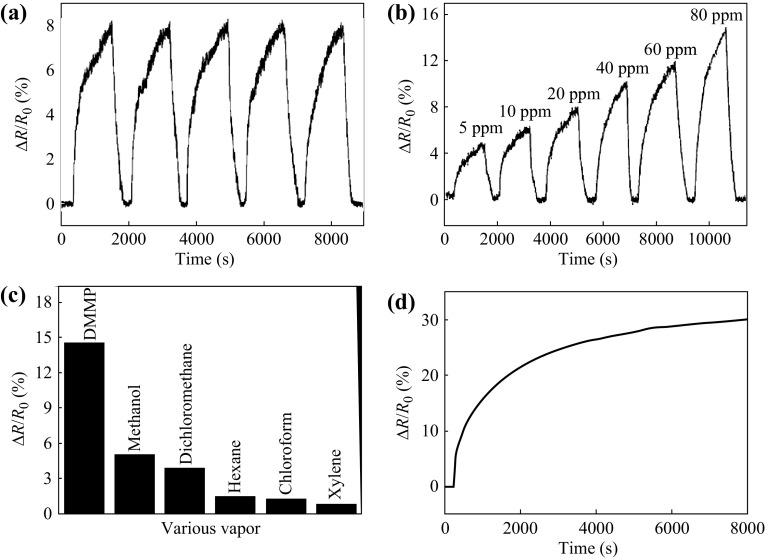
**a** Reproducibility of response of the RGO sensor to 20 ppm DMMP vapor. **b** Response curve of the RGO sensor to DMMP vapor under the concentrations of 5–80 ppm. (**c**) Response of RGO sensor to DMMP compared with other analytes diluted to 5 % of saturated vapor concentrations. **d** Response curve of the RGO sensor to DMMP vapor under the concentration of 80 ppm. Adapted from reference [63]

Through real-time monitoring and analyzing the response curves of sensing devices, the realistic realization of vapor detection can be achieved. Figure 5 is an example of real-time response of dimethyl methylphosphonate (DMMP) vapor monitored by para-phenylene diamine-reduced graphene oxide (PPD-RGO)-based vapor sensor. In Fig. 5, the excellent repeatability, low limit of detection, and superior selectivity of the vapor sensor have been distinctly displayed.

For evaluating the performance of gas/vapor sensors, there are a few critical parameters including component resistance, measure resistance, sensitivity, limit of detection, response time, recovery time, and selectivity. The definitions and formulas of these parameters are summarized in Table 1.

**Table 1:** Summary of the definition and formula of sensor parameters

Parameter	Definition	Formula
*R*_a_	Resistance value of the device, when put into the dry, clean atmosphere	
*R*_g_	Resistance value of the device, when put into gas to be detected	
*S*	Ratio of variation of resistance ($\left| {R_{\text{a}} - R_{\text{g}} } \right|$) to initial resistance (*R*_a_)	$S = \left| {\frac{\Delta R}{{R_{\text{a}} }}} \right| \times 100\,\% = \left| {\frac{{R_{\text{a}} - R_{\text{g}} }}{{R_{\text{a}} }}} \right| \times 100\,\%$
LOD	The lowest concentration of target gas that can be distinguished from the common atmosphere, which produces a signal greater than three times the standard deviation of the noise level	
*T*_res_	Period of time from gas sensor contact with gas to be detected to variation of resistance reach to 90 % of $\left| {R_{\text{a}} - R_{\text{g}} } \right|$	
*T*_rec_	Period of time from gas sensor away from gas to be detected to variation of resistance reach to 90 % of $\left| {R_{\text{a}} - R_{\text{g}} } \right|$	
*D*	Ratio of response of target gas (*S*_*c*_) to response of disturbed gas (*S*_*i*_).	$D = \frac{{S_{c} }}{{S_{i} }}$

## Synthesis and Properties of Graphene

There are mainly four approaches to synthesize single-layered or few-layered graphene: micromechanical exfoliation, epitaxial growth, vapor deposition, and chemical reduction [64–67]. Novoselov et al. used scotch tapes to repeatedly peel flakes of graphite off the mesas which were fixed onto a SiO_2_/Si substrate, and the high-purity, single-layered graphene was obtained [15]. By micromechanical exfoliation of highly ordered pyrolytic graphite, crystalline graphene nanosheets with large surface areas and a small number of layers could be obtained [65]. This method is very simple and does not need any special facilities. However, it is limited to laboratory research because of the small size and inefficiency of the production. Berger and his co-workers got graphene thin films which exhibited remarkable two-dimensional (2D) electron gas behaviors through thermal decomposition on the (0001) surface of 6H-SiC [68]. Epitaxial growth, compared with mechanical exfoliation, can realize the preparation of graphene with larger sizes and higher qualities. Hence, this approach is of significant importance for graphene semiconductor devices. Although a great breakthrough has been made for this technique, there is still a long way to go toward mass production of the graphene with uniform thickness and acceptable cost. Chemical vapor deposition (CVD) is the most extensively used method in industrial manufacture considering the merits of controllable sizes and structures. By pyrolysis of carbon-containing compounds, graphene was grown on the surfaces of transition metals, such as Cu [36], Pt [69], Ni [37], Ru [70], and Ir [71]. Copper foil is the most common substrate material to build single-layered graphene. Li and his group have successfully synthesized large-area and uniform graphene films on copper foils with a high quality by CVD techniques using methane as carbon source [36].

**Fig. 6:**
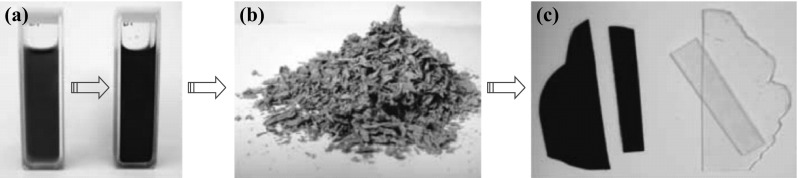
**a** Suspensions of phenyl isocyanate-treated graphite oxide (1 mg mL^−1^) and dissolved polystyrene in DMF before (*left*) and after (*right*) reduction by N, N-dimethylhydrazine. **b** Composite powders as obtained after coagulation in methanol. **c** Hot-pressed composite (0.12 vol% of graphene) and pure polystyrene of the same 0.4-mm thickness and processed in the same way. Adapted from reference [21]

In 2006, Stankovich et al. created a bottom-up approach when they incorporated graphene sheets in a composite material and the far-reaching method, which called chemical reduction of graphene oxide, pave the way for graphene’s large-scale production, modification, and application [21]. Figure 6 displays the fabrication process flow of graphene–polymer composite. In 2009, Tung et al. reported a versatile solution-based process for the large-scale production of single-layered chemically converted graphene over the entire area of a silicon/SiO_2_ wafer [72]. In general, there are three steps to obtain graphene-based composites: (1) strong oxidant, like H_2_SO_4_, HNO_3_, or HClO_4_, is used to transform graphite to graphite oxide. (2) complete exfoliation of graphite will take place, and molecular-level dispersion of individual graphene oxide (GO) in water or other polar solvent via ultrasonication will be achieved. (3) through the reduction of GO suspended in water or organic solvents, reduced graphene oxide (RGO) can be prepared without changing its morphology. Conductivity of RGO would be partly recovered too. The RGO sheets have quite high specific surface areas, which can be considered as a promising candidate for gas detection.

Brodie method [73], Staudenmaier method [74] and Hummers method [75] are three main ways to form GO. Hummers method is becoming the most popular approach to synthesize GO by virtue of its merits, including rapid, easy and relatively safe properties. Various modified Hummers methods have been reported to promote the progress of GO preparation [76–79].

In a sense, the development of chemical reduction can provide equivalent routes for production and modification of graphene materials via wet chemical techniques. As such, the reductant is so important since it can affect the properties of vapor detecting devices to a large degree [80–111]. Fan and co-workers observed that a stable graphene suspension could be quickly prepared by simply heating an exfoliated-GO suspension under strongly alkaline conditions at moderate temperatures (50–90 °C). This interesting reaction provides a green route to the synthesis of graphene with excellent dispersibility in water [81]. Zhu and his team developed a green and facile approach to synthesize chemically converted graphene nanosheets (GNS) through reducing exfoliated GO precursors by reducing sugars, such as glucose, fructose, and sucrose. Their unremitting efforts pave a new way to enlarge the production of widely used GNS with a high quality [95]. Recently, Liu et al. had demonstrated a green and facile approach to synthesize RGO through reduction of GO by Zn powder under acidic condition at room temperature. This approach offers a possibility for the production of RGO with cost-effective, environment-friendly and large-scale characteristics [106].

Recently, we found that PPD-reduced RGO exposed to DMMP exhibited much better response than that of the RGO reduced from hydrazine [63]. At the same time, we confirmed that RGO reduced from aniline exhibited a better response to ammonia, compared with the RGO reduced from hydrazine [107]. The sensing properties of aniline-reduced graphene attached with different states of polyaniline (PANI) had also been studied. The results suggested that free RGO exhibited better response to NH_3_ and showed higher sensitivity with concentrations at ppm levels compared to those of the RGO attached with acid-doped PANI and de-doped PANI [108].

## Properties of Gas/Vapor Sensors

Graphene has shown excellent sensing properties toward NH_3_, NO_2_, H_2_, CO, SO_2_, H_2_S, and volatile organic compounds (VOCs). Subsequently, some information from related works was summarized and discussed. Efforts have been made to exploit these sensitivities in the development of new sensor technologies.

### Ammonia Detection

Ammonia (NH_3_) is a compound of nitrogen and hydrogen with the formula NH_3_, which is a colorless gas with a characteristic pungent smell. Ammonia not only contributes significantly to the nutritional needs of terrestrial organisms by serving as a precursor to food and fertilizers, but also is a building-block for the synthesis of many pharmaceuticals, and is used in many commercial products. Although widely used, this gas is both caustic and hazardous, and thus it is harmful to human and would pollute environment. Therefore, the detection of NH_3_ is a pressing requirement for the modern society.

**Fig. 7:**
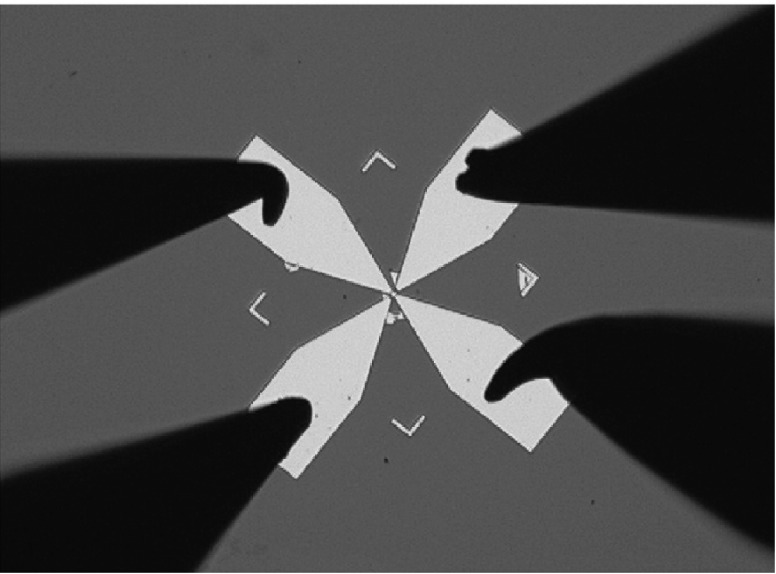
Optical micrographs of graphene film grown by CVD on Cu and then transferred onto a Si/SiO_2_ substrate. Gold contact pads in the Van Der Pauw configuration were deposited on the film. Adapted from reference [113]

**Fig. 8:**
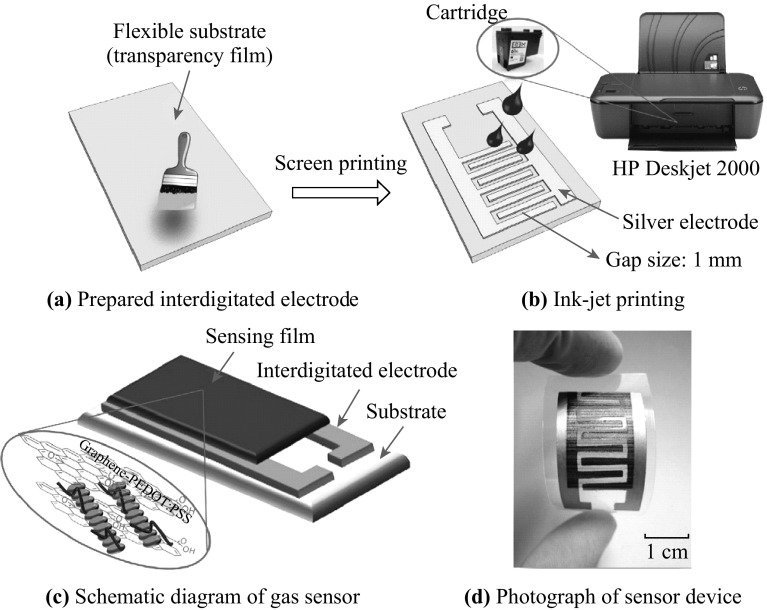
Schematic diagram of gas-sensor fabrication process. Adapted from reference [114]

Recently, a great deal of efforts had presented a great leap forward in the development of graphene gas sensors for ammonia detection. Gautam and his team investigated ammonia gas-sensing behaviors of graphene synthesized by CVD, of which the sensitivity and the recovery time were enhanced by the deposition of gold nanoparticles on the surface of graphene films [112]. Yavari et al. manufactured a device which was distinctly superior to commercially available NO_2_ and NH_3_ detectors [113]. They found graphene films synthesized by CVD (as displayed in Fig. 7) had an outstanding property of detection of NO_2_ and NH_3_ at room temperature. The detection limits of both NO_2_ and NH_3_ reached to ppb level. Wu and his co-workers reported a contrast experiment between graphene/PANI nanocomposites, and PANI to explore their sensing properties [61]. The results indicated that the NH_3_ detection limit of graphene/PANI sensors (ca. 1 ppm) was lower than that of PANI (ca. 10 ppm). This indicated that the sensitivity of graphene/PANI sensors for NH_3_ detection was enhanced by introduction of graphene into PANI. A simple, low-cost, and practical inkjet-printing technique for fabricating an innovative flexible gas sensor based on graphene–poly (3, 4-ethylenedioxythiophene):poly (styrene sulfonate) (PEDOT:PSS) composite films with high uniformity over a large area was created by Seekaew et al. [114]. Figure 8 clearly depicts a schematic diagram of this brand new gas sensor fabrication process. The ink-jet printed graphene-PEDOT: PSS gas sensor exhibited high response and high selectivity to NH_3_ in a low concentration ranging from 25 to 1000 ppm at room temperature. This novel and convenient method would provide a new thought for the controllable and mass manufacture of gas detectors. Table 2 summarized recent researches about NH_3_ detection based on graphene.

**Table 2:** A summary of recent researches about graphene-based gas sensors for NH_3_ detection at room temperature

Sensing material	Structure of sensor	Target gas	*T*_res_(s)	LOD	*T*_rec_(s)	Ref.
RGO/MnO_2_ + PANI	Chemiresistor	NH_3_	1080	25 %/5 ppm	240	[115]
RGO/ANI	Chemiresistor	NH_3_	1080	10.7 %/5 ppm	170	[107]
RGO/ANI + PANI	Chemiresistor	NH_3_	1080	20 %/20 ppm	120	[108]
RGO/Py	Chemiresistor	NH_3_	1.4	2.4 %/1 ppb	76	[116]
RGO/Py	Chemiresistor	NH_3_	720	4.2 %/50 ppb	375	[117]
GR + Au	Chemiresistor	NH_3_	1200	1 %/6 ppm	3800	[112]
GR	FET	NH_3_	–	0.49 V/ppm	–	[118]
GR	Chemiresistor	NH_3_	21,600	3 %/500 ppb	21,600	[113]
GR + PANI	Chemiresistor	NH_3_	50	0.7 %/1 ppm	23	[61]
GR supported by mica substrate	FET	NH_3_	60	4 %/50 ppm	–	[119]
GR gated by ionic liquid	FET	NH_3_	33	130 ppb	–	[120]
Printed GR + PEDOT:PSS	Chemiresistor	NH_3_	180	25 ppm	300	[114]
RGO + P3HT	Chemiresistor	NH_3_	141	7.15 %/10 ppm	488	[121]
RGO/Tannic acid	Chemiresistor	NH_3_	40	9.3 %/1310 ppm	170	[122]
RGO/Cu(OH)_4_^2−^+Cu_2_O	Chemiresistor	NH_3_	28	80 %/100 ppm	206	[123]

*RGO* reduced graphene oxide, *GR* Graphene, *PPD* p-phenyldiamine, *DMMP* dimethyl methyl phosphonate, *PANI* polyaniline, *ANI* aniline, *Py* pyrrole, *COP* Chemical oxidative polymerization

### Nitrogen Dioxide Detection

Nitrogen dioxide is one of several nitrogen oxides with the formula NO_2_. On one hand, this reddish-brown gas, as one kind of the important chemical feedstocks, is an intermediate in the industrial synthesis of nitric acid. On the other hand, the toxic gas has characteristic sharp, biting odor, and is a prominent air pollutant. The whole society has a strong demand for NO_2_ detection, in order to curb environmental pollution and keep the safety and health of human beings.

**Fig. 9:**
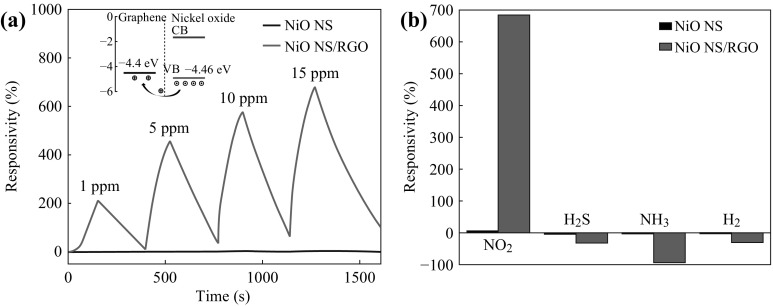
**a** Response of NiO nanosheet-based and NiO nanosheet/RGO-based gas sensors in various NO_2_ concentrations at 200 °C. **b** Response of NiO nanosheet-based and NiO nanosheet/RGO-based gas sensors in various gases, where the concentrations of NO_2_, H_2_S, and NH_3_ were 100 ppm, and H_2_ was 4 %. Adapted from reference [125]

**Fig. 10:**
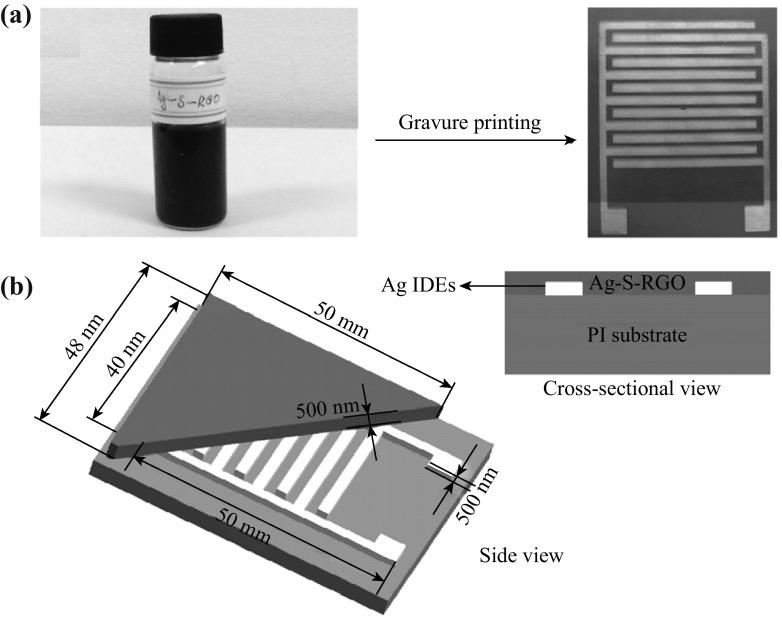
**a** Photographs of RGO/S + Ag ink and sensing layer printed onto the PI substrate with Ag-IDEs, respectively. **b** Schematic of the printed RGO/S + Ag sensor. Adapted from reference [126]

Compared to the development of ammonia detection, there are several reports about NO_2_ sensing showing the lower detection limit, higher response, and more practical manufacturing techniques. Choi and his co-workers reported a highly sensing NO_2_ gas sensor based on multilayered graphene films synthesized by a CVD method on a microheater-embedded flexible substrate [124]. The multilayered graphene had a very low detection limit of NO_2_ at sub-ppm (<200 ppb) levels. It also presented high responses and a short response time, when it was exposed to 1 ppm NO_2_ at room temperature. Hoa et al. reported that they built a gas sensor with hybrid structures of 2D graphene and 2D NiO nanosheets, sensitivity of which was two orders higher than those of devices based on NiO nanosheets alone toward NO_2_ even at 1 ppm level [125]. As shown in Fig. 9, the detector had excellent sensing properties, such as high sensitivity and superior selectivity. Nanosphere-like α-Fe_2_O_3_-modified RGO nanosheets were prepared by Dong’s team [109]. The 3D-structured nanocomposites exhibited a very high response of 150.63 % to 90 ppm NO_2_ at room temperature, which was 65.5 times higher than that of pure graphene, and the detection limit could be decreased down to 0.18 ppm. Huang et al. fabricated a gravure-printed chemiresistor-type NO_2_ sensor based on sulfonated RGO decorated with Ag nanoparticles (RGO/S + Ag) (as depicted in Fig. 10) [126]. Compared with other graphene-based sensors, this device showed more rapid response to NO_2_. When exposed to 50 ppm NO_2_, the sensor exhibited a sensitivity of 74.6 %, a response time of 12 s, and a recovery time of 20 s. Recently, Ju et al. reported a bendable and washable electronic textile (e-textile) gas sensors composed of reduced graphene oxides using commercially available yarns and molecular glues through an electrostatic self-assembly method [127]. The resultant e-textile gas sensor possessed the following features: (1) chemical durability to several detergents washing treatments, (2) mechanical stability under 1000 bending tests at an extreme bending radius of 1 mm, and (3) a high response to NO_2_ gas at room temperature with selectivity to other gases such as acetone, ethanol, ethylene, and CO_2_. Herein, we summarized recent researches about graphene-based gas sensors for NO_2_ detection, as shown in Table 3.

**Table 3:** A summary of recent researches about graphene-based gas sensors for NO_2_ detection at room temperature

Sensing material	Structure of sensor	Target gas	*T*_res_(s)	LOD	*T*_rec_(s)	Ref.
GR	Chemiresistor	NO_2_	3000	4 %/100 ppb	3000	[113]
Single-layered GR	FET	NO_2_	3600	2.5 ppm	–	[128]
Ozone-treated GR	Chemiresistor	NO_2_	900	1.3 ppb	1800	[129]
GR/PMMA on a flexible PET substrate	Chemiresistor	NO_2_	170	25 %/200 ppm	–	[130]
RGO/hydrazine + WO_3_	Chemiresistor	NO_2_	–	5 ppm	–	[131]
Multilayered GR	Chemiresistor	NO_2_	1800	6 %/1 ppm	–	[124]
RGO + NiO	Chemiresistor	NO_2_	125	200 %/1 ppm (200 °C)	250	[125]
Bilayer GR	FET	NO_2_	–	Establish a theoretical model	–	[62]
RGO/FeCl_3_ + α-Fe_2_O_3_	Chemiresistor	NO_2_	80	180 ppb	44	[109]
RGO + PVP	QCM	NO_2_	–	20 ppm	–	[132]
Printed RGO/S + Ag	Chemiresistor	NO_2_	12	74.6 %/50 ppm	20	[126]
RGO/hydrazine + ZnO	Chemiresistor	NO_2_	165	25.6 %/5 ppm	499	[133]
RGO + SnO_2_ aerogel	Chemiresistor	NO_2_	190	50 ppm	224	[134]
GO + Cs	Chemiresistor	NO_2_	240	90 ppb	540	[135]
RGO/NaBH_4_	Chemiresistor	NO_2_	420	11.5 %/5 ppm	1680	[136]
RGO + SnO_2_	Chemiresistor	NO_2_	75	3.31 %/5 ppm (50 °C)	300	[137]
RGO/WO_3_	Chemiresistor	NO_2_	540	769 %/5 ppm	1080	[138]
RGO/In_2_O_3_	Chemiresistor	NO_2_	240	8.25/30 ppm	1440	[139]

*RGO* reduced graphene oxide, *GO* Graphene oxide, *GR* Graphene, *PVP* Polyvinylpyrrolidone, *QCM* quartz crystal microbalance

### Hydrogen Detection

While hydrogen (H_2_) is not very reactive under standard conditions, it does form compounds with most elements. As one of the most important industrial chemicals and potential clean energy facing the future, hydrogen has aroused a great attention. Large-scale preparation, transportation, and application of this material have a strong demand for rapid detection and accurate analysis, which makes H_2_ detection become a research hotspot recent years.

**Fig. 11:**
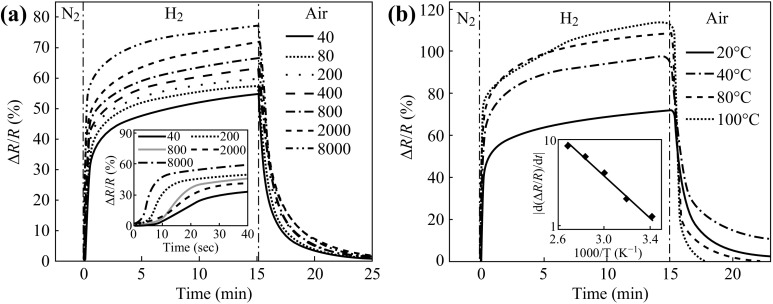
**a** The responses of the Pd-functionalized MLGN network sensor as a function of time when it is exposed to different concentrations of H_2_ in N_2_ ranging from 40 to 8000 ppm. **b** The response as a function of operating temperature in the range 20–100 °C for the MLGN network sensor when exposed to 2000 ppm H_2_. Adapted from reference [140]

**Fig. 12:**
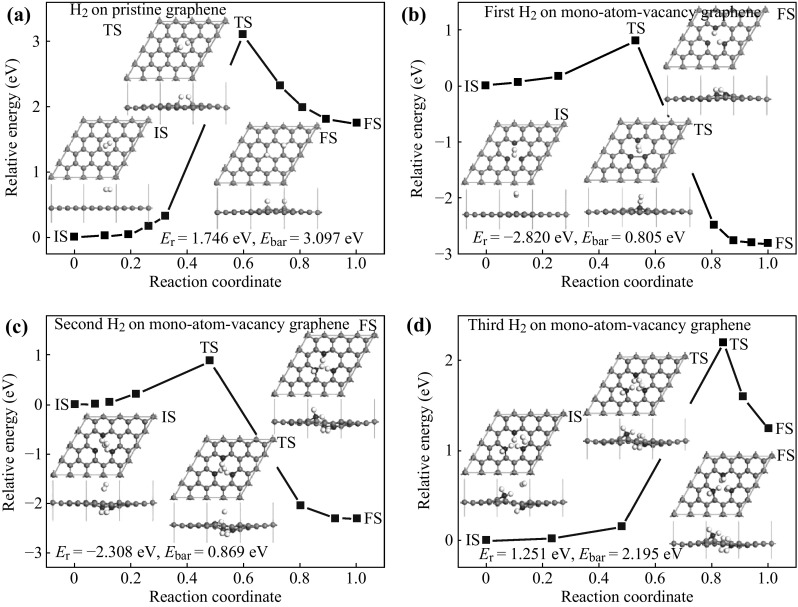
The reaction pathways of H_2_ molecule dissociative adsorption on pristine graphene (**a**), and on graphene with a mono-atom-vacancy for the first H_2_ molecule (**b**), the second H_2_ molecule (**c**), and the third H_2_ molecule (**d**). IS, TS, and FS represent initial structure, transition structure, and final structure, respectively. Their atomic structures are shown in the inserts. The energy of the IS is taken to be zero. The units of *E*_bar_ and *E*_r_ are eV, where *E*_bar_ is the energy barrier, and *E*_r_ is the reaction energy. The *gray*, *black*, and *white* atoms are saturated C, unsaturated C, and H, respectively. Adapted from reference [144]

Johnson and his co-workers reported a novel Pd-functionalized multilayered graphene nanoribbon networks with excellent sensitivity to H_2_ at ppm levels. The fluffy porous material structure and noble metal modification accounted for their fast response and recovery time at room temperature [140]. The relationship between the sensor performance and work temperature was studied as well. Their work offers the possibility of using functionalized graphene-based nanoribbon networks in a wide range of gas/vapor-sensing applications. Figure 11 shows the response of the device varying with the concentration of H_2_ and work temperature. The real-time response curves of the detector as well as the activation energy of hydrogen detection at background temperatures varied from room temperature to 175 °C were measured by Chu et al. [141]. Three *E*_a_ (activation energy) were observed dependent on the background temperature: 0.832 eV for 30–60 °C, 0.396 eV for 60–100 °C, and 0.057 eV for 100–170 °C. Their results contribute to the theoretical research of gas/vapor detection. Meanwhile, Chu and his team studied the effect of thickness of the Pt metal layer on hydrogen-sensing sensitivity of Pt-coated and multilayered graphene, and they concluded that the Pt coating improved the response time of the graphene sensor, but decreased the sensitivity [142]. When the thickness of the Pt metal layer was about 1 nm, the sensor presented the highest sensitivity. Mehta and co-workers had successfully fabricated a device with ultrafast response and recovery of hydrogen sensing based on graphene composite layers with Pd and Pt nanoparticles dispersed on graphene layers [143]. Jiang et al. considered the dissociative adsorption of H_2_ molecules on graphene with mono-atom-vacancies by using density functional theory (DFT) calculations [144]. They demonstrated that this defected graphene was promising for ultrasensitive room-temperature hydrogen sensing and the LOD could even reach to 10^−35^ mol L^−1^ theoretically. The reaction pathway of H_2_ molecule dissociative adsorption on pristine graphene and treated graphene with a monoatom-vacancy was displayed in Fig. 12. Table 4 summarized recent researches about H_2_ detection based on graphene.

**Table 4:** A summary of recent researches about graphene-based gas sensors for H_2_ detection at room temperature

Sensing material	Structure of sensor	Target gas	*T*_res_(s)	LOD	*T*_rec_(s)	Ref.
Pt/RGO/SiC	FET	H_2_	300	Voltage shift of ≈100 mV for 1 % H_2_ (100 °C)	–	[145]
GR/Pt	Chemiresistor	H_2_	540	16 %/4 vol%	–	[140]
Multilayered GR/Pd nanoribbon	Chemiresistor	H_2_	21	55 %/40 ppm	23	[146]
GR/Pt	Chemiresistor	H_2_	700	1 % concentration (175 °C)	700	[141]
GR/Pt	Chemiresistor	H_2_	120	80 %/1 % concentration	1200	[142]
GR/(Pt + Pd)	Chemiresistor	H_2_	<2	2 % concentration (40 °C)	18	[143]
GR/Pd	Chemiresistor	H_2_	–	1 % concentration	–	[147]
GR/Pd	Chemiresistor	H_2_	900	20 ppm	1800	[148]
GR	First-principle calculation	H_2_	–	–	–	[149]
RGO/TiO_2_/(Pd + Pt)	Chemiresistor	H_2_	18	92 %/500 ppm (180 °C)	29	[150]
RGO/SnO_2_ + Pt	Chemiresistor	H_2_	5	1 % concentration	4	[151]
RGO/Pd	Chemiresistor	H_2_	–	0.20 %	–	[152]
GR with mono-atom-vacancy	First-principle calculation	H_2_	–	10^−35^ mol L^−1^	–	[144]
RGO/Pd	Chemiresistor	H_2_	1200	0.4 %/0.2 ppm	900	[153]
GO	Chemiresistor	H_2_	270	6 %/800 ppm	306	[154]
PMMA/Pd NPs + SL GR	Chemiresistor	H_2_	108	66 %/2 %	330	[155]
GR/SnO_2_ NPs	FET	H_2_	1.2	3/100 ppm	1.6	[156]
GO/PEDOT:PSS	Chemiresistor	H_2_	30	4.2 %/100 ppm	25	[157]

*RGO* reduced graphene oxide, *GR* Graphene, *PMMA* Polymethylmethacrylate, *NPs* Nanoparticles, *SL* Single layer

### Carbon Dioxide, Carbon Monoxide, and Methane Detection

Carbon dioxide (CO_2_), carbon monoxide (CO), and methane (CH_4_) are very familiar to our daily life, industrial manufacture, and environmental protection. CO_2_ is not only the primary source of carbon in life, but also of significant impact in air pollution, which can cause global warming. CO is toxic to humans when encountered in concentrations above about 35 ppm. Besides, this colorless, odorless, and tasteless gas is one kind of gaseous fuels, which is widely used as reducing agent in industry. CH_4_ is the simplest alkane and the main component of natural gases. On one hand, the relative abundance of methane makes it an attractive fuel. On the other hand, it is the chief culprit of a gas explosion. In a word, detection and early warning of these gases is a pressing need for modern society.

Nemade et al. have carried out a lot of work focusing on graphene-based carbon dioxide sensor over the recent years [158]. They fabricated a device with excellent stability, short response and recovery times, and low detection limit based on few-layered graphene synthesized by an electrochemical exfoliation method. It is worth mentioning that this few-layered graphene also showed remarkable sensing features to liquid petroleum gases, which endowed it with a giant potential application. In addition, they investigated the sensing characteristic to CO_2_ of graphene/Y_2_O_3_ quantum dots (QDs) [159], graphene/Sb_2_O_3_ QDs [160], and graphene/Al_2_O_3_ QDs [161], respectively. The experimental results showed that gas-sensing properties could be changed by different combination of materials. Liu and his team investigated the adsorption of several common gas molecules (CO, SO_2_, NH_3_, CO_2_, N_2_, H_2_O, and H_2_) on Li-decorated T graphene, using DFT [162]. They found that Li-decorated T graphene exhibited a higher sensitivity to CO. Their work provided an insight to build promising gas detectors based on graphene. Wu et al. reported that graphene nanosheets/PANI nanocomposite with a different mass ratio was synthesized and investigated [163]. This hybrid was able to fabricate methane sensor, the detection limit of which decreased with the increasing mass ratio of graphene to PANI. Herein, we summarized recent researches about graphene-based gas sensors for CO_2_, CO and CH_4_ detection, as shown in Table 5.

**Table 5:** A summary of recent researches about graphene-based gas sensors for CO_2_, CO, and CH_4_ detection at room temperature

Sensing material	Structure of sensor	Target gas	*T*_res_(s)	LOD	*T*_rec_(s)	Ref.
GR/PANI	Chemiresistor	CH_4_	85	10 ppm	45	[163]
GR/Li	First-principle calculation	CO	–	–	–	[162]
GR prepared by mechanical cleavage	Chemiresistor	CO_2_	8	9 %/10 ppm	–	[164]
GR/Y_2_O_3_ QDs	Chemiresistor	CO_2_	–	1.08 %/35 ppm	–	[159]
Few-layered GR	Chemiresistor	CO_2_	11	3 ppm	14	[158]
GR reduced by hydrogen plasma	Chemiresistor	CO_2_	240	2 %/300 ppm	240	[165]
GR/Sb_2_O_3_ QDs	Chemiresistor	CO_2_	16	50 ppm	22	[160]
GR/Al_2_O_3_ QDs	Chemiresistor	CO_2_	14	100 ppm (125 °C)	22	[161]

*GR* Graphene, *QDs* quantum dots

### Sulfur Dioxide and Hydrogen Sulfide Detection

As main atmospheric pollutants, sulfur dioxide (SO_2_) and hydrogen sulfide (H_2_S) are very harmful to mankind and animals. In recent years, some researchers reported some novel gas sensors for the detection of SO_2_ and H_2_S based on graphene composites. Shen and his team demonstrated that GO nanosheets derived from chemically tailoring acted as a promising material for SO_2_ gas sensing [166]. The edge-tailored GO nanosheet-based chemiresistive sensor had a wide range of sensitivity as well as a quick response and short recovery time at room temperature. First principle calculations based on DFT were often used to predict the physical properties of specific materials. Through DFT calculation, Liu et al. drew a conclusion that Al-doped defective graphene owned a high reactivity toward SO_2_, indicating its potential application in SO_2_ detection [167]. Similarly, Shao et al. found that Cr-doped zigzag graphene nanoribbons were also considered as the potential candidates for SO_2_ molecular sensors [168]. Tensile strain effects on enhanced adsorption of H_2_S molecules on Ag-decorated defective graphene composite were investigated using first principles calculations based on DFT by Xian’s team [169]. Their calculations illustrated that a relatively modest tensile strain around 8 % in defective graphene can greatly increase the binding energy of Ag adatom by 44 %, indicating enhanced stabilization of Ag adatom on defective graphene, while the tensile strain had little effects on the sensitivity of Ag-decorated defective graphene composite to H_2_S molecule. Zhou et al. fabricated a RGO/Cu_2_O nanocomposite-based sensor with a very low detection limit of 5 ppb at room temperature, which might be on account of high surface activity adsorption of H_2_S gas molecules due to the absence of any surfactant capping [170]. So far, it is the lowest LOD in the similar types of sensors. Jiang and co-workers had also carried out a fantastic work to realize ultrafast response to H_2_S within 500 μs, as well as a fast recovery time of less than 30 s [171]. They used magnetic fields with different orientations to control fabrication progress of the Fe_2_O_3_/graphene nanosheets. The experimental results illustrated that structural orientation of nanosheets played an essential role in maximizing efficiency of the device. In a word, their remarkable jobs and significant results have greatly promoted the development of graphene-based gas sensors. Table 6 summarized recent researches about SO_2_ and H_2_S detection based on graphene.

**Table 6:** A summary of recent researches about graphene-based gas sensors for SO_2_ and H_2_S detection at room temperature

Sensing material	Structure of sensor	Target gas	*T*_res_(s)	LOD	*T*_rec_(s)	Ref.
GR	FET	SO_2_	120	100 %/50 ppm	120	[172]
Edge-tailored GO	FET	SO_2_	–	5 ppm	–	[166]
Al-dropped defective GR	First-principle calculation	SO_2_	–	–	–	[167]
Cr-doped zigzag GR nanoribbons	First-principle calculation	SO_2_	–	–	–	[168]
Ag-decorated defective GR	First-principle calculation	H_2_S	–	–	–	[169]
Ag-supported Si-doped GR	First-principle calculation	H_2_S	–	–	–	[173]
Fe-dropped defective GR	First-principle calculation	H_2_S	–	–	–	[174]
RGO + Cu_2_O nanocrystal	Chemiresistor	H_2_S	120	11 %/5 ppb	120	[170]
PSS-doped RGO/PANI	Chemiresistor	H_2_S	<90	1 ppm	150	[175]
RGO/SnO_2_ NFs	Chemiresistor	H_2_S	<198	1 ppm (200 °C)	<114	[176]
RGO/Fe_2_O_3_	Chemiresistor	H_2_S	500 μs	15 ppm (190 °C)	<30	[171]
GR/porous WO_3_ NFs	Chemiresistor	H_2_S	–	3.9 %/100 ppb (300 °C)	600	[177]
Zigzag Gr/Cu	First-principle calculation	H_2_S				[178]
GR/Ti or GR/Sn	First-principle calculation	SO_2_/H_2_S				[179]

*RGO* reduced graphene oxide, *GO* Graphene oxide, *GR* Graphene, *PSS* poly 4-styrenesulfonic acid, *NFs* Nanofibers

### Volatile Organic Compounds, Explosives, and Chemical Warfare Agents Detection

Volatile organic compounds (VOCs) are organic chemicals that have a high vapor pressure at room temperature. VOCs are numerous, varied, and ubiquitous. They refer to gases which containing organic compounds, including aromatic hydrocarbon, nitro hydrocarbon, halogenated hydrocarbon, long chain alkane, alcohol, ether, acetone, grease, hydrazine, and so on. Most of them are toxic, flammable, and explosive gases. At present, as the terrible activities are of high frequency, the detection of explosives and chemical warfare agents (CWAs) attracts an increasing attention in many fields and is becoming a hot topic for research.

In general, the study of graphene-based vapor sensors for detection of VOCs, explosives, and CWAs is relatively immature. As such, many novel approaches have been developed to explore the terra incognita.

Dua and co-works developed a rapid and one-step method for the conversion of exfoliated GO into RGO using aqueous vitamin C as a mild and green reducing agent [180]. The RGO-based gas sensor fabricated by inkjet printing techniques was able to detect VOCs at ppb level at room temperature. In 2011, Jiang et al. developed a facile and novel route to synthesize Al_2_O_3_/graphene nanocomposites with the aid of supercritical CO_2_ derived from graphene oxide [181]. The ethanol-sensing features of as-synthesized Al_2_O_3_/graphene nanocomposites were firstly reported on the basis of catalytic chemiluminescence mechanisms. They boldly broke through the limitation of the traditional preparation and measurement methods, leading a new way to tackle relevant problems. In the same year, Zhang et al. reported an intrinsic polymer optical fiber (POF) sensor based on graphene, which was described for the purpose of acetone vapor sensing for the first time [182]. Gautam’s team had systematically studied the key parameters (response, recovery, repeatability and reliability) of the sensor based on gold and platinum nanoparticles functionalized graphene for the detection of different organic vapors (acetic acid, ethanol, and acetone) at ppm levels [183].

Tang et al. established a prominent analytical platform for electrochemical sensing determination of nitroaromatic explosive compounds, such as 2,4,6-trinitrotoluene (TNT), which was superior to other TNT-sensing platforms, using uniform and rich-wrinkled graphene films prepared by electrophoretic deposition techniques [184]. The detection of TNT with the concentration of 0.2 ppb in a phosphate buffered saline by differential pulse voltammetry was realized. Fan’s team utilized water-soluble and surface-unmodified graphene quantum dots, which were prepared by a chemical approach from GO, as a novel, effective, and simple fluorescent-sensing platform for ultrasensitive detection of TNT in solution by fluorescence resonance energy transfer quenching for the first time [185]. The detection limit was about 0.495 ppm. Liu et al. used surface enhanced Raman scattering to realize ultratrace detection of TNT (5 × 10^−16^ M), which was based on p-aminothiophenol functionalized graphene nanosheets decorated with silver nanoparticles [186]. GO modified Au electrode was used as a carbon electrode catalyst for the electrochemical oxidation of chemical warfare agent simulant thiodiglycol (TDG) at room temperature by Singh and his team [187]. Their experiments indicated that GO would be a better alternative material for transition metals in the degradation of chemical warfare agents as well as environmental pollutants. Ganji et al. drew a conclusion that aluminum nitride graphene had stronger interaction with the DMMP molecule and could provide more sensitive signal for a single DMMP molecule, compared with pristine graphene, boron nitride graphene, using ab initio van der Waals density functional calculations [188]. Though some detection process of their experiments could only take place in solution, their excellent work is a useful reference for graphene-based gas detection and has contributed a lot to practical applications in national defense and daily life. Herein, we summarized recent researches about graphene-based vapor sensors for VOCs, explosives, and CWAs detection, as shown in Table 7.

**Table 7:** A summary of recent researches about graphene-based vapor sensors for VOCs, explosives, and CWAs detection at room temperature

Sensing material	Structure of sensor	Target gas	*T*_res_(s)	LOD	*T*_rec_(s)	Ref.
RGO/PPD	Chemiresistor	DMMP	1080	5 %/5 ppm	360	[63]
Few-layered GR	Chemiresistor	LPG	5	4 ppm	18	[158]
RGO/SnO_2_ NFs	Chemiresistor	Acetone	<198	100 ppb (350 °C)	<114	[176]
GO/Au electrode		TDG				[187]
Al nitride GR	First-principle calculation	DMMP				[188]
Uniform and rich-wrinkled GR		TNT		0.2 ppb		[184]
GQDs	FRET quenching	TNT		0.495 ppm		[185]
GR/Ag + PATP	SERS	TNT		5 × 10^−16^ M		[186]
Printed RGO	Chemiresistor	VOCs		ppb level		[180]
RGO/Al_2_O_3_	CL	Ethanol	10	1.5 mg/mL^−1^ (200 °C)	<100	[181]
GR on POF	OFS	Acetone		44 ppm		[182]
RGO	FET array	Ethanol	300	17 %		[189]
GR/(Au + Pt)	Chemiresistor	VOCs		30 %/100 ppm		[183]
GO/PPr	Chemiresistor	Toluene		24 ppm		[190]
Ni NPs/Nafion/GR	CV & EIS	Ethanol		0.12 mM		[191]
RGO/ZnFe_2_O_4_	Chemiresistor	Acetone	4	10 ppm (275 °C)	18	[192]
Si dropped BC_3_ GR	First-principle calculation	Acetone				[193]
Self-Assembled GR/PDA	Colorimetric sensor	VOCs		0.01 %		[194]
Co_3_O_4_ NFs + Ir NPs + GO	Chemiresistor	Acetone		1.18 %/120 ppb (300 °C)		[195]
RGO coated optical fiber	OFS	Methanol & Ethanol		100 ppm		[196]
RGO/Ag	OFS	Ethanol	11	1 %	6	[197]
RGO/ZnO + Ag NPs	Chemiresistor	Acetylene	21.2	21.2/100 ppm (150 °C)	80	[198]
RGO/ZnO + Ag NPs	Chemiresistor	Acetylene	57	12.3/100 ppm (200 °C)	90	[199]

*RGO* reduced graphene oxide, *GO* graphene oxide, *GR* graphene, *TDG* thiodiglycol, *GQDs* graphene quantum dots, *FRET* fluorescence resonance energy transfer quenching, *PATP* p-aminothiophenol, *SERS* surface enhanced Raman scattering, *CL* catalytic chemiluminescence, *POF* polymer optical fiber, *PPr* polypyrene, *NPs* nanoparticles, *CV* cyclic voltammetry, *CIS* electrochemical impedance spectroscopy, *PDA* polydiacetylene, *NFs* Nanofibers, *OFS* optical fiber sensor

## Response Mechanisms

We have given a brief introduction to the classification of gas/vapor sensors. Considering that the gas-sensing mechanisms of graphene is uncertain and related research is rare, herein, we just give a recognized point of view as a general introduction of the reference of other related literatures [200–203].

Graphene is intrinsically inert and nonselective. Its great efficiency to conduct electricity and distinguishing features of ballistic transport of charges decide that this two-dimensional material is an ideal candidate to serve as a platform or a supporter, in which we can realize many specific functions by doping or compositing with other materials. Once combined with other materials physically or chemically, graphene can show the characteristics of the semiconductor in normal circumstances, of which conductivity is determined by carriers’ concentration. For chemiresistor-type sensors, sensing materials show response to externalities by the change of conductivity, that is the variation of concentration of hole or electron carriers. Bulk porous materials usually have a large specific surface area, hence gas molecules can be easily adsorbed, following by the interaction between gas molecules and specific groups in the graphene surface, and then the gas molecules capture or donate electrons from the sensing material, which changes concentration of the semiconductor’s carriers.

**Fig. 13:**
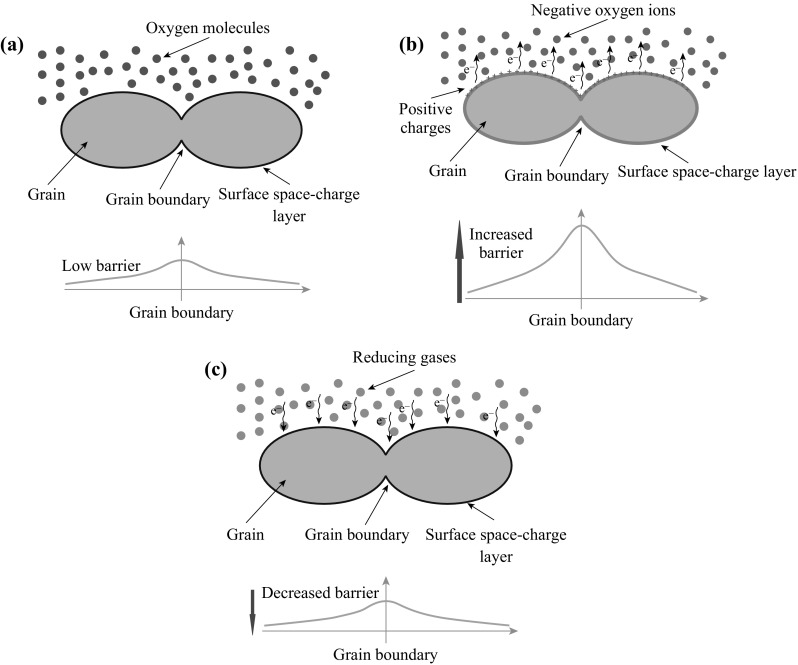
A general progress of gas sensing based on chemiresistor

Different doping and reaction conditions may lead to different types of graphene-based semiconductors (p-type or n-type). As we all know, p-type semiconductors refer to those who have a larger hole concentration than electron concentration. In p-type semiconductors, holes are the majority carriers and electrons are the minority carriers. As opposed to p-type semiconductors, n-type semiconductors have a larger electron concentration than hole concentration. In n-type semiconductors, electrons are the majority carriers and holes are the minority carriers. For example, one doped graphene shows characteristic of n-type semiconductors: when it is exposed to a reducing atmosphere, such as NH_3_, it would get electrons from the gas molecules, leading to an increase of the electron concentration, i.e., a decrease of graphene’s resistance occurs. Likewise, when it is exposed to an oxidation atmosphere, such as NO_2_, it will deliver electrons to the gas molecules, leading an increase of hole concentration, leading to an increase of graphene’s resistance. Figure 13 demonstrates a general progress of gas sensing, which has been described above. This is the old and universal theory called “Oxygen anion barrier model,” which used to illustrate the mechanism of gas-sensing progress based on metal-oxide semiconductors [204–207].

Zhou et al. have found that the total flow rate had a significant effect on the initial electric resistance of the sensors and their sensing properties to target gases. In addition, an appropriate quantity of deposited RGO solution was critical for sensors’ sensing response and sensitivity. Finally, they raise a novel sensing mechanism for chemiresistors based on RGO at room temperature [208]. Zhu and his team had done an important job to prove that the oxygen functional groups presiding on the surface of reduced graphene oxide could play a vital role in the response for one specific gas. Two types of unprecedented effects could be attributed to the presence of oxygen functional groups, i.e., the selective binding interactions (strong or weak) to different gas molecules, and the impendence to charge interaction between gas molecules and sp2-hybridized carbon areas in RGO [209]. Sometimes, p-type graphene and n-type graphene can transform from one to another by changing the annealing temperature. Wang et al. explored this interesting phenomenon that the slightly reduced p-type graphene showed ultrasensitive gas sensing at room temperature, with a response of 58 % to 1 ppm ethanol, while the graphene could become n-type and insensitive to gas sensing, with a low response of 0.5 % to 50 ppm ethanol, by simply increasing the annealing temperature to about 300 °C [210].

## Conclusions

### Existing Problems

The interests in the study of nanomaterials have escalated in the recent decades, while the application is still in its infancy. This so-called “game changing” technology has met, one after another, many impediments on its way to large-scale industrialization [40]. Can graphene and graphene-based devices get through the close siege?

Theory can indicate a direction for practice. However, till now, the mechanism of gas sensing based on nanomaterials is not very clear, and quantitative calculation is almost impossible. There is little doubt that graphene thin film has great sensitivity; however, this may lead to another result that it is sensitive to many kinds of gases. Cross-sensitivity means sensor shows similar responses to the different types of gases, and this character may result in false detecting. For example, cross-sensitivity can be a problem in the detection of ethylene oxide, as ethylene oxide requires a very active working electrode catalyst and high operating potential for its oxidation. Therefore, gases which are more easily oxidized like alcohols and carbon monoxide will also give a response. Once a technique reaches the stage of mass production, it will be a completely different compared with the laboratory. One of dire challenges we confronted with is the nonrepeatability of device fabrication. From preparation of sensing materials to construction of gas/vapor sensors, from building of experimental platforms to characterization parameters, none of the uniform criteria is listed, and neither specification of laboratory equipment nor the unified presentation of technological process and synthesis method was reported.

### Solutions and Prospects

In order to overcome the problems mentioned above, we put forward several worthwhile schemes and directions. Sensing materials are the core of gas detection in a real-world application. The development of synthesizing novel materials with high sensitivity and selectivity is one of the mainstream trends of gas sensors. Multicomponent classification and hybrid nanostructures which have multifunctions and outstanding performances in practical tasks are at the forefront of current research. By the improved preparation techniques such as modification of graphene, 3D structure tailoring, and thermal treatments, we may make sensors to suit the ideal state.

**Fig. 14:**
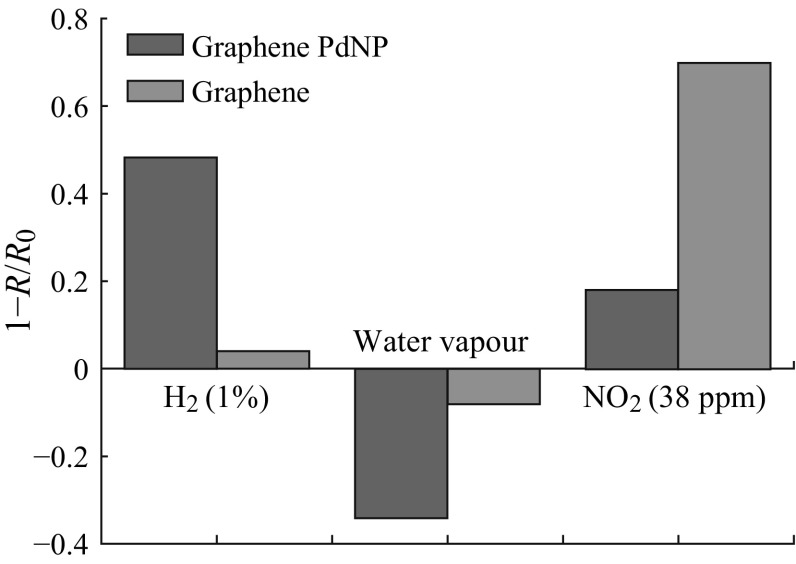
Comparison of the responses to hydrogen, NO_2_, and humidity of the graphene/Pd NPs composite and of graphene. Adapted from reference [147]

**Fig. 15:**
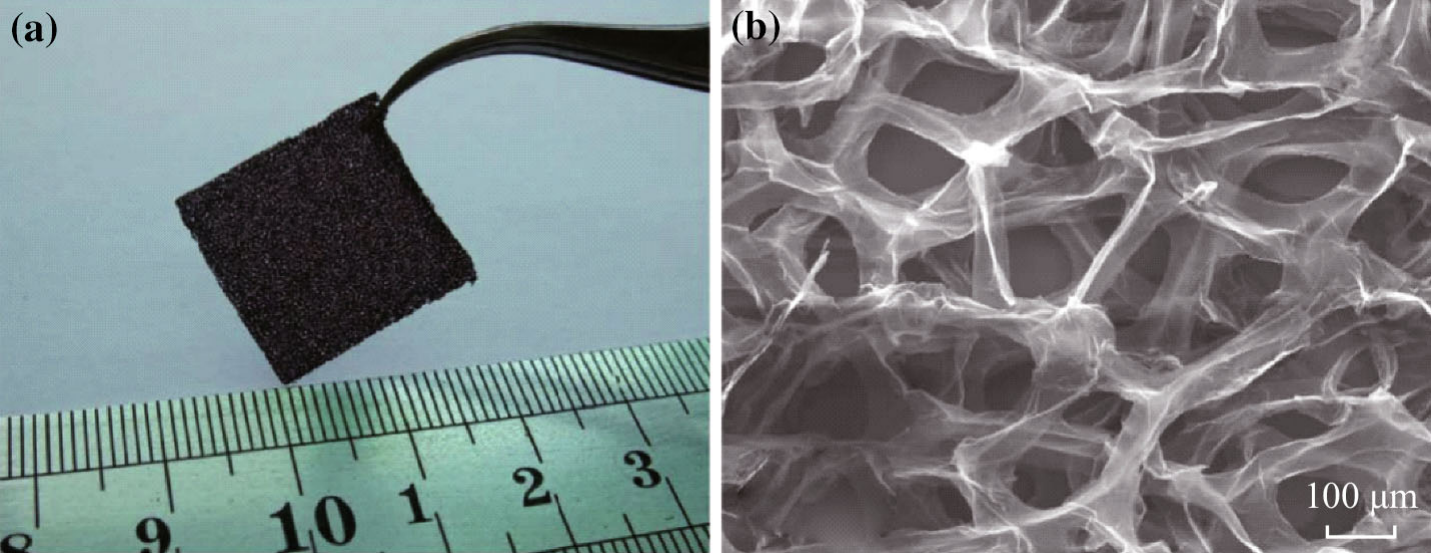
**a** Photograph and **b** scanning electron micrograph of the microporous graphene foam structure showing a continuous network of 3D interconnected graphene sheets that comprise the walls of the foam-like structure. The robust and flexible graphene foam strips can be easily handled and manipulated. Adapted from reference [211]

Figure 14 shows a stark contrast between the responses of the graphene/palladium nanoparticle composites to H_2_, NO_2_, and humidity and those of pristine graphene. The comparison explicitly instructs that modification can change the sensing properties to a large extent. Yavari and his team manufactured a macro-graphene foam-like 3D network which had both the advantages of the nanostructured and conventional solid-state and conducting-polymer sensors. A surprising sensing property and ppb level detection of NH_3_ and NO_2_ in air at room temperature had been demonstrated for this robust, flexible, and novel material [211]. The microporous structure of this graphene foam is demonstrated in Fig. 15.

**Fig. 16:**
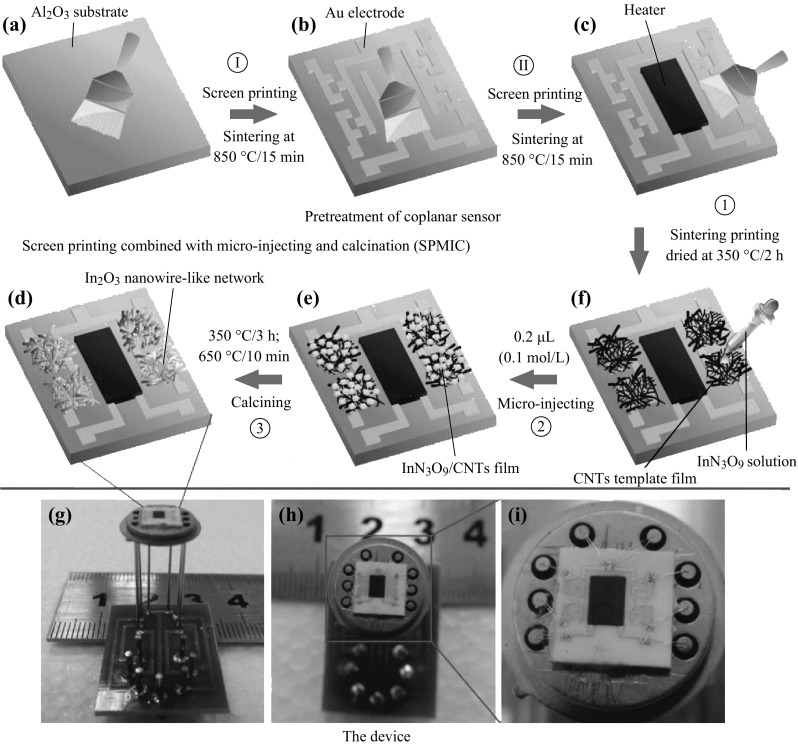
Schematic diagram of the preparation of sensing material and the construction of the device. Adapted from reference [212]

Based on the technology of microelectromechanical systems, multiple sensor arrays, in which every unit has different heterostructure and shows different sensing characteristics, can be assembled and expected to have higher sensitivity and improved selectivity. Yi et al. presented a novel materials—sensor integration fabrication strategy, which involved the introduction of micro-injection to fabricate sensing devices. The In_2_O_3_ nanowire-like network directly on the surface of coplanar sensors array by structure replication from sacrificial CNTs was obtained on the basis of screen-printing technology and calcination. The device showed that excellent gas-sensing properties benefited from fabrication of coplanar gas sensors arrays and materials, which had special porous nanowire-like network micromorphology. Figure 16 shows a schematic diagram depicting the procedure to prepare the porous In_2_O_3_ nanowire-like network and the related devices.

**Fig. 17:**
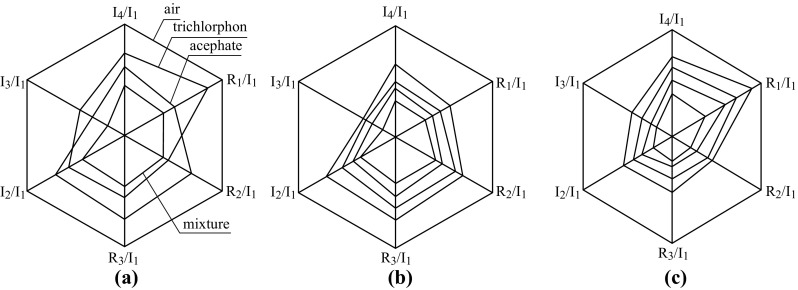
**a** Characteristic responses of the relative intensities of the higher harmonics. *R*_*i*_ and *L*_*i*_ are the real and imaginary components of the *i*th higher harmonic. The analyzed data correspond to the resistance values in the concentrations of 1.053 μg L^−1^ trichlorphon, 0.75 μg L^−1^ acephate, and their mixture. **b**, **c** Effects of concentrations of pesticide vapor on the relative intensities of the higher harmonic. The concentrations from inside to outside are 0.75, 2.25, 3.75, and 5.25 μg L^−1^; and 1.053, 3.159, 5.265, and 7.371 μg L^−1^, respectively. **b** acephate, **c** trichlorphon. Adapted from reference [213]

The employment of the new signal-processing technology and recognition algorithm based on single-chip system is an important direction for the development of gas-sensing devices. By the application of dynamic detection, signal processing, and recognition algorithm, gas/vapor sensors with low power consumption, portable volume, and intelligent operation could be achieved [213–215]. Huang and his team had successfully achieved qualitative and quantitative analysis of organophosphorus pesticide residues using temperature-modulated SnO_2_-based gas sensor, and the quantitative analyses of the pure pesticide vapor and their mixture were performed by fast Fourier transformation [213]. The results showed that the amplitudes of the higher harmonics exhibited characteristic changes depending on the vapor concentration ratio and the kinetics on the sensor surface, as shown in Fig. 17. They made a significant exploratory development in the rapid detection of pesticide residue vapors.

The future of graphene-based gas/vapor sensors looks bright. Continued progress in this field will overcome the current challenges, get through the close siege, and lead to a class of gas sensors with superior sensitivity, excellent selectivity, reduced size, and extended lifetimes for a wide range of environments and applications.
